# Pupil size and social vigilance in rhesus macaques

**DOI:** 10.3389/fnins.2014.00100

**Published:** 2014-05-06

**Authors:** R. Becket Ebitz, John M. Pearson, Michael L. Platt

**Affiliations:** ^1^Department of Neurobiology, Stanford University School of MedicineStanford, CA, USA; ^2^Department of Neurobiology, Duke University School of MedicineDurham, NC, USA; ^3^Department of Evolutionary Anthropology, Duke UniversityDurham, NC, USA

**Keywords:** social vigilance, pupil size, pupil light response, distractibility, task performance, social attention

## Abstract

Complex natural environments favor the dynamic alignment of neural processing between goal-relevant stimuli and conflicting but biologically salient stimuli like social competitors or predators. The biological mechanisms that regulate dynamic changes in vigilance have not been fully elucidated. Arousal systems that ready the body to respond adaptively to threat may contribute to dynamic regulation of vigilance. Under conditions of constant luminance, pupil diameter provides a peripheral index of arousal state. Although pupil size varies with the processing of goal-relevant stimuli, it remains unclear whether pupil size also predicts attention to biologically salient objects and events like social competitors, whose presence interferes with current goals. Here we show that pupil size in rhesus macaques both reflects the biological salience of task-irrelevant social distractors and predicts vigilance for these stimuli. We measured pupil size in monkeys performing a visual orienting task in which distractors—monkey faces and phase-scrambled versions of the same images—could appear in a congruent, incongruent, or neutral position relative to a rewarded target. Baseline pupil size under constant illumination predicted distractor interference, consistent with the hypothesis that pupil-linked arousal mechanisms regulate task engagement and distractibility. Notably, pupil size also predicted enhanced vigilance for social distractors, suggesting that pupil-linked arousal may adjust the balance of processing resources between goal-relevant and biologically important stimuli. The magnitude of pupil constriction in response to distractors closely tracked distractor interference, saccade planning and the social relevance of distractors, endorsing the idea that the pupillary light response is modulated by attention. These findings indicate that pupil size indexes dynamic changes in attention evoked by both the social environment and arousal.

## Introduction

Attention prioritizes portions of the local environment for enhanced neural processing. The stimuli prioritized by attention are often relevant to current goals. Nevertheless, biologically relevant stimuli, such as the faces of social partners and competitors, can attract attention despite conflict with current goals. While the reflexive deployment of attention to biologically relevant stimuli can facilitate threat detection and prioritize social behavior, it also interferes with pursuit of any goal that requires sustained attention, such as foraging. Attentiveness to biologically relevant stimuli that compete with sustained goal pursuit is known as “vigilance” in ethology (Lazarus, 1978; Pöysä, 1994; Roberts, 1996; Hunter and Skinner, 1998; Hirsch, 2002). In nature, vigilance is dynamically regulated in response to changes in the local environment including the likelihood of predation (Hunter and Skinner, 1998; Hirsch, 2002) and neighbor proximity (Lazarus, 1978; Pöysä, 1994; Roberts, 1996). The biological mechanisms that regulate vigilance state remain poorly understood, particularly for social cues.

Norepinephrine (NE) is one likely regulator of vigilance. NE acts on both the central and peripheral nervous system, and is responsible for activation of the sympathetic nervous system in response to threat. How NE contributes to vigilance, particularly in social contexts, remains unclear. One possibility is that NE regulates vigilance by adjusting the balance of attention devoted to goal pursuit (Aston-Jones and Cohen, 2005; Yu and Dayan, 2005; Eldar et al., 2013). Consistent with this idea, NE tone, as indexed by the spiking rates of neurons in the locus coeruleus—the brainstem source of central NE—varies with arousal state and performance on attention demanding tasks (Foote et al., 1980; Rajkowski et al., 1994; Aston-Jones and Cohen, 2005). Another commonly used peripheral index of NE tone is pupil size under constant luminance (Samuels and Szabadi, 2008; Gilzenrat et al., 2010; Jepma and Nieuwenhuis, 2011; Nassar et al., 2012; Eldar et al., 2013). Under these conditions, pupil size predicts learning (Nassar et al., 2012), an effect that may be mediated by alterations in attention allocated to task-relevant stimuli (Eldar et al., 2013). NE could thus affect vigilance by regulating task engagement.

However, NE may also have effects on attention to goal- or task-irrelevant stimuli. There is limited and contradictory pharmacological evidence in support of this hypothesis. Ablation of the ascending NE system increases distractibility (Carli et al., 1983) but agonists of the inhibitory alpha-2 autoreceptor, which decrease NE tone, suppress distractibility (Clark et al., 1989; Witte and Marrocco, 1997). Moreover, the effects of alpha-2 antagonists on distractibility are dependant on individual variation in baseline distractibility (Bunsey and Strupp, 1995). Additionally, it remains unclear whether variations in NE levels within the normal physiological range predict distractibility. This is a significant gap because while the pharmacological effects appear to be non-linear, a linear relationship between NE and distractibility has long been hypothesized to exist at physiologically typical levels (Aston-Jones et al., 1999; Aston-Jones and Cohen, 2005). Moreover, physiologically typical NE tone has an inverted u-shaped relationship with other functions, such as working memory (Arnsten, 2009) and task performance (Aston-Jones et al., 1999; Aston-Jones and Cohen, 2005). Finally, it remains unclear whether increasing NE levels predict a truly labile state of attention, which may not be an adaptive response to heightened arousal, or instead a more specific and adaptive response like the promotion of a species-typical vigilance state.

Though pupil size varies with NE tone under constant luminance, the primary job of the pupil is to adjust the amount of light entering the eye in response to changes in luminance. The most obvious example of this is the pupil light response, a rapid and largely reflexive constriction of the pupil in response to a transient luminance increment. Intriguingly, the pupil light response is not completely determined by luminance but also varies with task performance (Steinhauer et al., 2000), stimulus awareness during binocular rivalry (Hakerem and Sutton, 1966; Zuber et al., 1966), threat of shock (Bitsios et al., 1996), pharmacological manipulations of NE (Bitsios et al., 1998), and instructions to attend to a bright stimulus (Binda et al., 2013a). Transient pupil constriction also follows isoluminant changes in visual stimuli (Barbur et al., 1992; Kardon, 1995; Sahraie and Barbur, 1997; Gamlin et al., 1998) that attract attention. The onset of coherent motion, for example, both captures attention (Abrams and Christ, 2003) and evokes transient pupil constriction (Barbur et al., 1992; Sahraie and Barbur, 1997).

One possible explanation for these observations is that the pupil light response scales with stimulus attention. However, previous studies have only measured the pupil light response to task-relevant stimuli. Attention to task-relevant stimuli is conflated with other factors known to affect pupil size such as effort and task engagement. However, stimulus attention can be functionally dissociated from task engagement or effort by examining attention to task-irrelevant distractors, rather than to task-relevant stimuli. Effort, task engagement, and task-relevant stimulus attention all improve task performance. However, task-irrelevant stimulus attention hinders task performance through increasing the interference of distractors. Thus, if the pupil response to distractors scales negatively with distractor interference, it would suggest that it is effort or task engagement, rather than stimulus attention, which modulates the pupil light response. Conversely, if the pupil light response to distractors scales positively with the distractors' task interference, it suggests that the pupil light response is modulated by stimulus attention, beyond any effect of effort or task engagement.

To test these hypotheses directly, we probed pupil size in rhesus macaques while they performed a visual orienting task in which biologically salient faces competed for attention with rewarded targets. Rhesus macaques and humans possess remarkably similar oculomotor systems and have pupil light responses mediated by homologous neural pathways (Clarke et al., 2003). Moreover, the relationship between activity in the locus coerruleus—the source of NE in the brain—and pupil size has only been demonstrated in the rhesus macaque (Gilzenrat et al., 2010). Faces attract gaze at the expense of competing goals in both humans (Cerf et al., 2009) and rhesus macaques (Ebitz et al., 2013) in the absence of any systematic training or instructions, indicating that both species are spontaneously vigilant for this biologically salient class of stimuli. The development of a behavioral model of vigilance in the rhesus macaque is an important first step toward characterizing the local neural circuits and neuromodulatory mechanisms that regulate vigilance state, permitting invasive measures and manipulations that are only possible in an animal model.

We found that increasing baseline pupil size at trial onset predicted increasing interference of distractors. This provides indirect support for long-standing hypotheses regarding the relationship between NE and task performance (Aston-Jones and Cohen, 2005). Moreover, baseline pupil size also predicted enhanced interference of social distractors relative to non-social distractors, suggesting that pupil-linked arousal states may specifically modulate vigilance for biologically salient environmental cues in addition to non-specific changes in alertness and focus. This finding accords with the idea that increasing NE tone increases attentional deployment to those stimuli which were already most likely to be attended (Eldar et al., 2013). We also found that the magnitude of the pupil response to light varies with the spatial locus of attention, trial-to-trial variation in the effects of distractors on response time, the social significance of distractors, and pre-saccadic processes. While baseline pupil size predicted both distractor interference and the magnitude of the pupil light response, the pupil light response itself varied systematically with distractor interference even after controlling for baseline pupil size. These findings thus indicate that dynamic changes in attention scale with changes in the pupil light response, suggesting a shared underlying process. This observation endorses the idea that higher-level attentional processes are closely integrated with lower-level light control mechanisms in natural vision. Together, these observations indicate that pupil size signals two partially distinct components of vigilance and thus provides a powerful tool for understanding the dynamic expression and regulation of vigilance.

## Methods

### Behavioral techniques

All techniques were approved by the Duke University Institutional Animal Care and Use Committee (protocol A011-12-01). Using standard techniques (Hayden et al., 2008), four male rhesus macaques were surgically-prepared with head restraint prostheses under isoflurane anesthesia to permit high-resolution infrared videography of eye position and pupil size, as well as subsequent neurophysiological recording. Analgesics were used to minimize post-surgical discomfort. After recovery, the monkeys were placed on controlled access to fluids to motivate task performance. Data collection for this task began a minimum of 4 weeks post-operatively but in most cases occurred several months after surgery. A portion of the data presented here was collected in conjunction with electrophysiological recordings.

Eye position and pupil size were monitored at 1000 Hz via infrared eye tracking (SR Research; Eyelink). The manufacturer's standard center of mass (centroid) method was used to calculate both pupil direction and size. There is a possibility that some pupil size measurements may have been affected by occlusion of the pupil by the eyelid. Nevertheless, the experimenter monitored pupil size via visual inspection of the infrared camera during experimental sessions and did not observe any pupil occlusion during any trials in any of the monkeys. Moreover, any change in the occlusion of the pupil at the start of a trial would necessarily result in an inaccurate mapping between the monkey's veridical eye position and the eye tracker's estimate, prohibiting the initial acquisition of fixation necessary to begin the trial. Blinks were identified using the manufacturers' standard algorithm and trials with blinks were not included in the final analyses. Custom scripts written in Matlab using Psychtoolbox-3 were used to display stimuli and record eye position. Task stimuli were colored targets presented against a dark background on a 51 cm wide LCD monitor (60 Hz refresh rate, 1920 × 1080 resolution), located 60 cm from the monkey.

**Figure 1:**
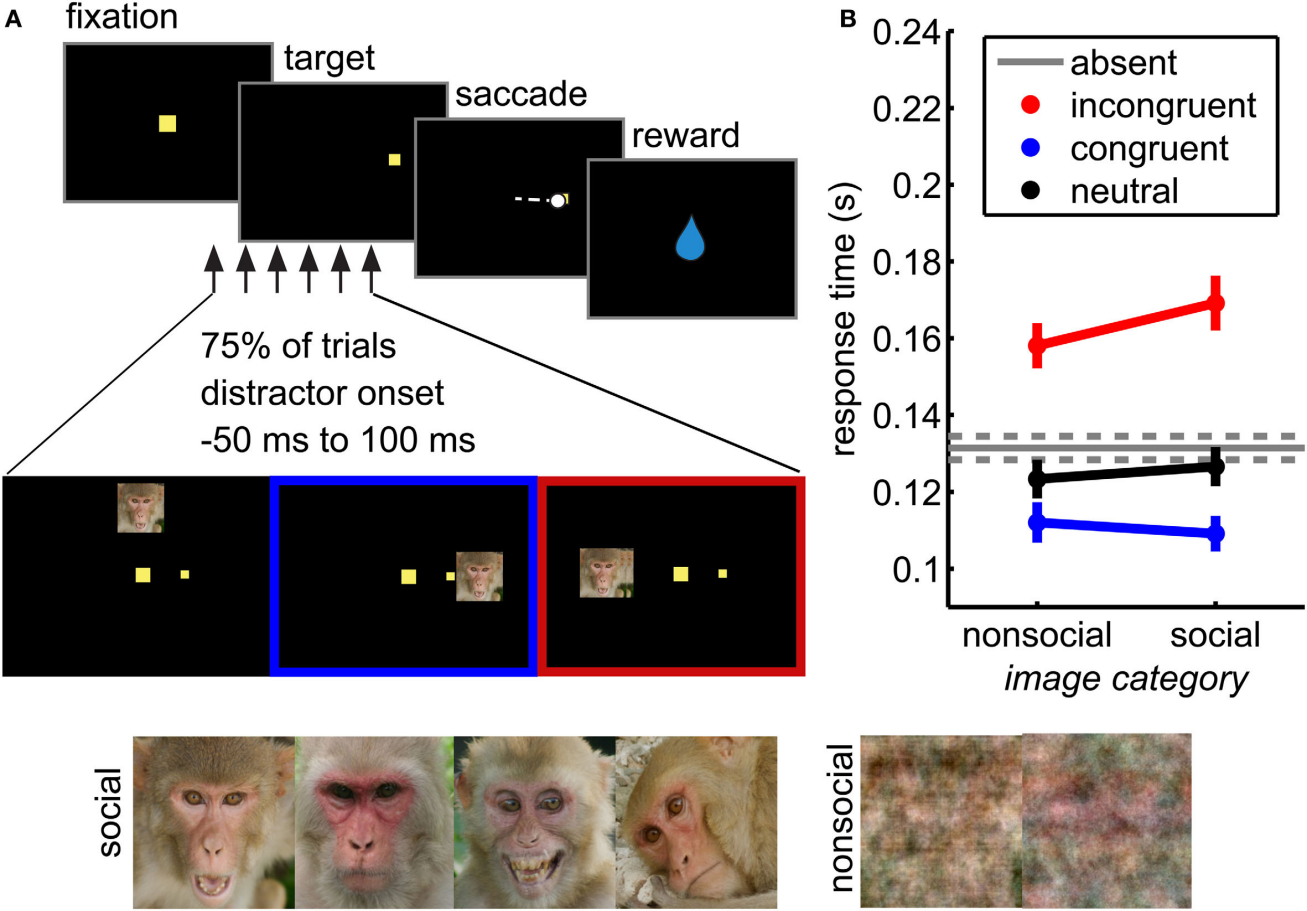
**Social distraction task. (A)** Distractors were briefly flashed (for 67 ms) during performance of a visually guided saccade task. These distractors were either social or non-social (phase scrambled) images. **(B)** Distractors interfered with response time in this task. Incongruent distractors (red) slowed responses, whereas congruent distractors sped responses (blue) relative to distractor absent trials (gray). Neutral distractors did not significantly affect response time (black). Social distractors magnified these response time effects (interaction of location and distractor image type, *F*_(3, 38684)_ = 10.81, *p* < 0.05 × 10^−5^). Bars ± s.e.m. across sessions.

The social interference task (Figure 1) is a visually guided saccade task with distractors. Monkeys first fixated a central 1° target (±6° of error) for 450–650 ms and then shifted gaze to an eccentric target (1° square) appearing either 14° left or right of the fixation stimulus. Fixation on the eccentric target (±6°) for 150–450 ms resulted in juice reward, the magnitude of which was fixed for each monkey within sessions and ranged from 0.15 to 0.35 mL per trial.

On a randomly chosen 75% of trials, a non-predictive distractor was briefly flashed for 67 milliseconds (the duration of 2 screen refreshes), the leading edge of which was 15° from the fixation stimulus, ensuring that it never overlapped the target position. Distractors were presented at one of three locations relative to the target: congruent (same hemifield), incongruent (opposite hemifield), or neutral (directly above fixation). Distractors were presented with a variable stimulus onset asynchrony (SOA) relative to target onset (50 ms before target onset to 100 ms after, uniformly and continuously distributed).

Distractors were large (7° wide) images of rhesus macaque faces or phase-scrambled versions of the same images. The face images (157 images) were drawn from a database of pictures of rhesus macaques on Cayo Santiago, Puerto Rico. The images were selected to maximize heterogeneity across genders, ages, emotional expressions, viewing angles, and gaze direction, although both eyes were visible in each image. The images were cropped to include a whole face and resized to a standard 248 × 248 pixel size. RGB images were converted to NTSC color space and then the luminance channel was adjusted to match mean luminance across all images. Control images (157 images) were generated by phase scrambling each resized and intensity-matched social images in MATLAB. The phase scrambling added identical randomly generated noise (from –pi to pi) to each Fourier-transformed color channel before recombining the images into RGB space, then converted to NTSC as above. Thus, social and control images were matched for overall intensity.

### Pupil measurements

The diameter of the pupil was sampled at 1000 Hz on an Eyelink II infrared eye tracker (SR Research), using the manufacturer's standard methods for calculating pupil area. Any occlusion of the pupil due to blinks was removed and trials on which blinks were detected during fixation were aborted. We investigated both baseline pupil diameter and the pupil light response. Baseline pupil diameter on each trial was calculated as the average diameter over all pupil size samples collected during the first 350 ms of fixation (350 samples). Pupil size was first locally averaged with a Gaussian kernel (8 ms standard deviation). The pupil light response was calculated as the peak percent change in pupil size in the 600 ms following distractor onset, measured relative to the first 50 ms. In some analyses, pupil size or pupil responses were binned by quantiles. In each of these analyses, the pupil measure was binned into 30 quantiles within each session. The figures show different numbers of bins for clarity, but the fits shown are from models run on 30 quantile bins, unless otherwise noted. In order to compare distractor-aligned pupil responses to trials in which distractors were absent, distractor absent trials were aligned to sham distractor time stamps. Sham distractor timestamps were drawn with replacement from the distribution of distractor time stamps on normal distractor trials.

### Data analysis

Data were analyzed in MATLAB. Standard receiver operating characteristic (ROC) analyses (MATLAB perfcurve) were used to determine discriminability between pupil constriction magnitudes on distractor present vs. distractor absent task conditions, as well as between distractor locations. Separate ROC curves were generated within each session and the range of areas under the curves (AUCs) across sessions is reported in the text. Within each session and across sessions, permutation tests were used to determine the significance of the AUCs. Labels were shuffled 500 times per session, producing 500 synthetic shuffled data sets in which each trial was randomly labeled as distractor or no-distractor. Thus, for each shuffled dataset, discriminability between the two conditions should be at chance. These shuffled datasets constitute a distribution for the AUC statistic under the null hypothesis of no pupil response difference between trial types. Within each session, the observed AUC was compared to the shuffled AUCs in a one-sided bootstrap test, at the significance threshold noted in the text. Across all sessions, a Wilcoxon rank sum was used to determine whether the observed AUCs differed from the shuffled AUCs across all sessions.

ANOVAs were mixed effects models that accounted for random main effects of monkey and session, with session nested within monkey. All other variables were treated as fixed effects nested within session. ANOVAs included all possible two-factor interaction terms. Paired *t*-tests were used in all *post-hoc* tests to compare within session means, unless otherwise noted, and corrected for multiple comparisons. ANOVAs were used to analyze the baseline response time (variables included distractor social content and trial type) and the effect of distractor presentation time on the pupil light response (variables included SOA and trial type).

All other analyses utilized generalized linear models, as described below. In addition to the terms included in the following equations, each model contained an error term to account for variation between monkeys. The first models were used to predict response time from both baseline pupil diameter and the magnitude of pupillary response to the distractor. Within each session, the pupil measure was binned into 30 quantiles, to allow comparisons across sessions, and mean response time was calculated within each pupil size bin for congruent and incongruent distractor trials. The following model was then run on the quantile-binned data.

$$RT=\beta_{0}+\beta_{1}\left(pupil\right)+\beta_{2}\left(\alpha \right)+\beta_{3}(\alpha )(pupil)$$

**Figure 2:**
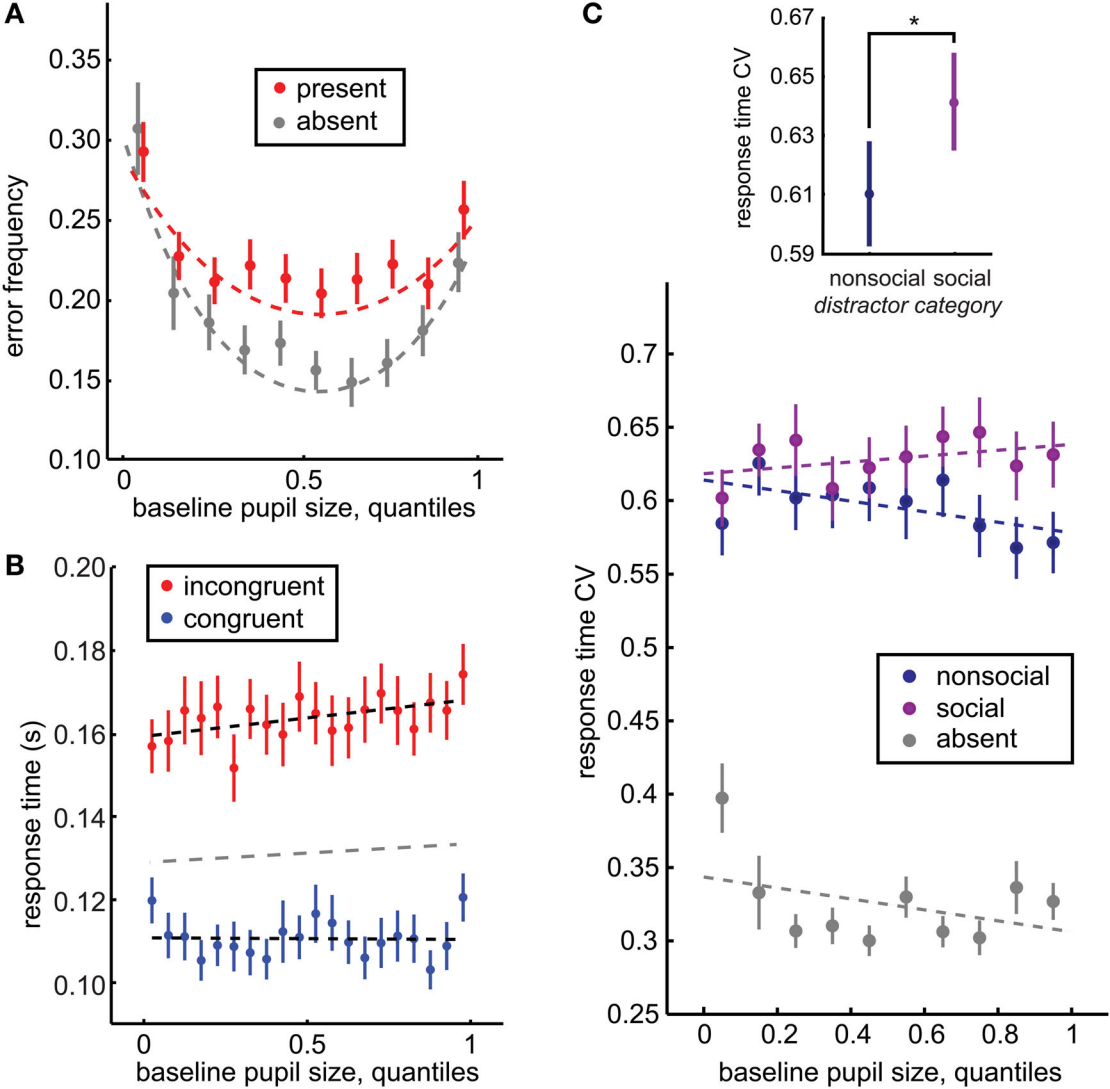
**Baseline pupil size predicts distraction by social stimuli. (A)** Baseline pupil size had a U-shaped relationship with error commission, regardless of whether distractors were present (red) or absent (gray). However, there was also an interaction between pupil size and distractor presence: increased pupil size predicted a specific increase in error commission in the presence of distractors (*p* < 0.0001, β_4_ = 0.055). **(B)** Baseline pupil size also predicted the increased difference between the response time effects of congruent (blue) and incongruent (red) distractors (significant interaction of distractor location and baseline pupil size *p* < 0.0003, β_3_ = 0.0008). A separate GLM fit to distractor-absent response time is plotted in gray. **(C)** The CV of response time is a measure of the dispersion of the response time distributions across the incongruent and congruent locations. Inset: Response time CV was larger following social distractors than non-social distractors, bars reflect ± standard deviation. Main figure: Response time CV is specifically enhanced for social distractors as baseline pupil size increases (*p* < 0.02, β_5_ = 0.059), suggesting that the increasing variance in response time with increasing baseline pupil size is driven by the social distractors. Bars ± s.e.m. across sessions. Dotted lines reflect GLM model fits to binned data (30 quantile bins; for clarity of visualization, a smaller number of bins is plotted in each panel). ^*^p < 0.02, z_(71)_ = 2.47.

**Figure 3:**
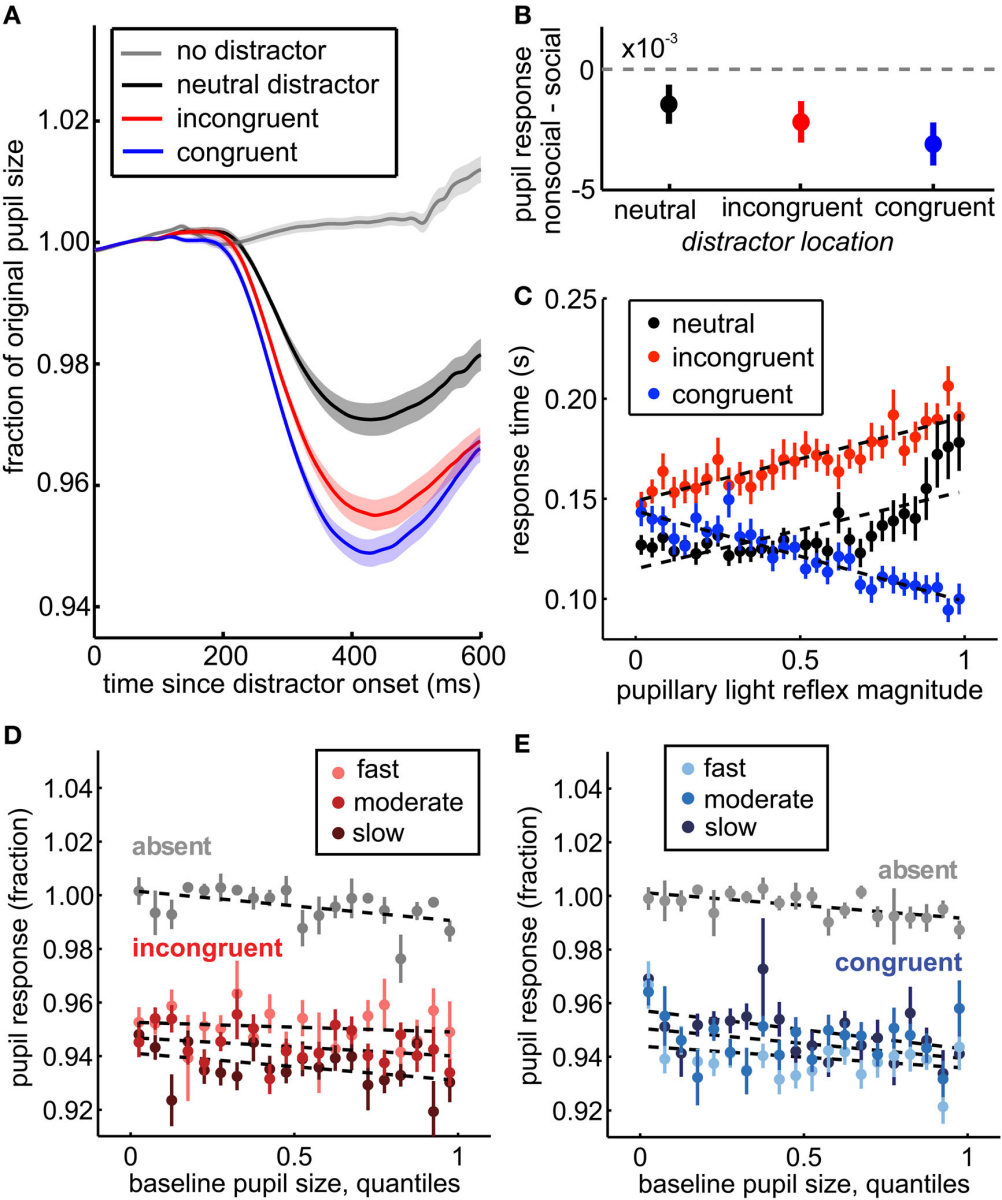
**The pupil light response indexes spatial attention, social relevance, and trial-by-trial variation in distractor response time effects. (A)** Pupil traces, averaged across sessions, aligned to distractor onset (black, red, and blue traces) or to sham distractor time stamps (gray). The pupil response was enhanced for distractors in congruent (blue) and incongruent (red) locations compared to distractors that did not bias response time (neutral distractors, black). No pupil response was observed in the absence of distractors (gray). Shading ± s.e.m. across sessions. **(B)** Greater pupil constriction was observed for social images than non-social images, regardless of whether they were spatially incongruent (red), congruent (blue), or neutral (black) with respect to the target. Bars ± s.e.m. across sessions. **(C)** Within distractor locations, the magnitude of pupil constriction after distractor onset predicted their response time effects. Larger pupil light responses predicted longer saccade reaction times on incongruent distractor trials (red) and shorter saccade reaction times on congruent distractor trials (blue). Neutral trials are plotted for comparison (black), but not included in the GLM. Dotted lines reflect fits from the GLM for incongruent and congruent trials, and a least squares fit for the neutral trials. **(D)** Baseline pupil size predicted a small, but significant shift in subsequent pupil size regardless of the presence of distractors (*p* < 0.01, β_1_ = −0.004). This shift did not explain the relationship between the pupil distractor response and response time, however. Instead, larger pupil light responses were still associated with slower response times following incongruent distractors (*p* < 0.0001, β_5_ = −0.056). Response time is divided into 3 equally spaced bins within session for illustration, though models were run on raw data. Faster responses are plotted in brighter colors relative to slower responses. **(E)** Same as **(D)** for congruent distractors. Baseline pupil size also predicted subsequent pupil light responses on congruent trials (*p* < 0.0001, β_3_ = 0.013), but larger pupil responses still predicted faster response times on congruent distractor trials when controlling for baseline pupil size (*p* < 0.0001, β_5_ = 0.042). Bars ± s.e.m. across sessions. Dotted lines reflect GLM model fits to binned data (30 quantile bins; for clarity of visualization, a smaller number of bins is plotted in each panel).

Where “pupil” was a vector of pupil size quantile bins and α was a logical vector with 1 for incongruent trials and 0 for congruent trials. β_1_ thus reflected the relationship between pupil size and congruent trials, β_2_ a constant offset between congruent and incongruent response times, and β_3_ the interaction effect of distractor congruency on response time: the relationship between pupil size and response time on incongruent trials, relative to congruent trials. Figures 2C, 3A reflect fits from this model.

In order to probe the relationship between baseline pupil size and the social relevance of distractors, the model was elaborated to include a third term to differentiate between social and non-social distractors.

$$\begin{array}{l} rtCV=\beta_{0}+\beta_{1}(pupil)+\beta_{2}(\alpha )+\beta_{3}(\gamma )+\beta_{4}(\alpha )(pupil)\\ \;+\beta_{5}(\gamma )(pupil) \end{array}$$

In this case α was 0 in the absence of distractors and 1 when they were present. γ was 1 for social distractor trials and 0 for non-social distractor trials. The dependent variable (rtCV) was the coefficient of variation in response times across congruent and incongruent distractor locations within each bin (standard deviation divided by mean). Similar effects were found when we calculated the CV across all distractor locations, however only distractors in the congruent and incongruent locations had appreciable effects on response time, so only these distractors were analyzed here. Response time CV was used because it has additional sensitivity to the variance in response times compared to a simple difference between the mean response times across distractor locations. Specifically, response time CV is sensitive to apparently incongruent changes in distractor interference (such as slowed target detection response times following highly salient congruent distractor images) and to changes in the interference of small numbers of distracting images from the larger set (such as selective changes to the images that typically have the largest attentional priority), which would have a larger effect on the variance of response times than the mean.

In order to determine whether variation in baseline pupil size explained the relationship between the pupil light response and distractor interference, we employed a GLM which included a term for baseline pupil size and allowed for interactions between baseline pupil size and response time bin in explaining the variance in the pupil response. The model was as follows:

$$\begin{array}{l} \Delta pupil=\beta_{0}+\beta_{1}(baseline)+\beta_{2}(\alpha )+\beta_{3}(\alpha )(baseline)\\ \;+\beta_{4}(\alpha )(RT)+\beta_{5}(\alpha )(RT)(baseline) \end{array}$$

“Δ*pupil*” refers to the pupil light response described previously. Baseline pupil size was zscored within sessions for this analysis and included as the term “size”. Raw response times were included as the term “RT.” Finally, the term “α” simply specified the presence (1) or absence (0) of distractors. This model was fit separately for congruent and incongruent trials. The fitted beta weights were interpreted as follows. β_1_ reflected the relationship between pupil size at fixation and pupil size in the time window following real or sham distractors, β_2_ reflected a constant offset between pupil response to real and sham distractors, β_3_ reflected the interaction of distractor presence and baseline pupil size, β_4_ reflected the offset between response time bins, and β_5_ captured any differences in slope between response time bins. For plotting, both baseline pupil size and distractor-present response times were divided into quantile bins, in order to allow comparisons across monkeys and sessions within a single figure. The same model was then run for illustrative purposes on the quantile-binned data in order to generate the model fits shown in Figures 3D,E.

In order to determine whether baseline pupil size predicted changes in the likelihood of errors (failures to saccade to the target) and errant saccades (saccades off fixation that were not directed toward the target), we used a third, quadratic model with a logistic link function.

$$\begin{array}{l} \ln\left(err/\left(1-err\right)\right)=\beta_{0}+\beta_{1}\left(\alpha \right)+\beta_{2}\left(pupil\right)+\beta_{3}\left(pupil^{2}\right)\\ \;+\beta_{4}\left(\alpha \right)\left(pupil\right)+\beta_{5}\left(\alpha \right)\left(pupil^{2}\right) \end{array}$$

**Figure 4:**
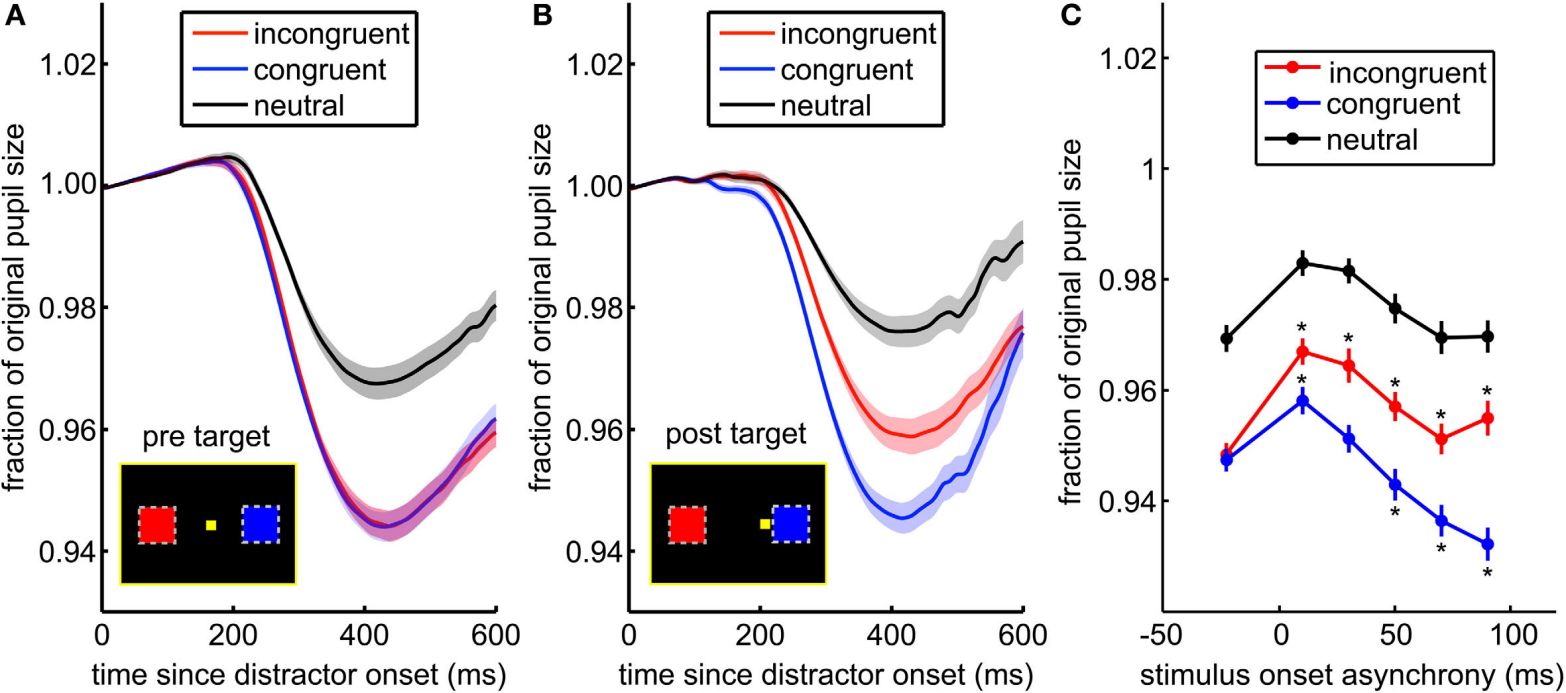
**The pupil light response is enhanced by saccade planning. (A)** When the distractor was presented before the target appears, there was no difference between the pupil response to incongruent (blue) and congruent distractors (red). **(B)** When distractors were presented after the target, the pupil response distinguished between congruent and incongruent distractors. Shading ± s.e.m. **(C)** This effect is due to both suppression of the response to distractors at non-target locations (incongruent distractors in red), and enhancement of the response to distractors at locations proximate to the upcoming saccade (congruent distractors in blue). Asterisks (^*^) mark post-target distractor presentation times where the pupil response was either significantly greater than the pre-target response (^*^ above the data point) or less than the pre-target response (^*^ below the data point).

We combined occurrences of errors (trials in which reward was not received because of broken target fixation or failure to saccade to the target within the specified window) and errant saccades (trials in which reward was received, but the initial saccade off fixation was not directed toward the target) for this analysis. Baseline pupil size was binned by within-session quantiles into 30 bins, which were used as the “pupil” regressor. The term “α” specified the presence (1) or absence (0) of distractors. This squared term in this model accounted for the U-shaped relationship we observed between pupil size and error likelihood (Figure 4B).

We used a Bayesian Information Criterion approach to select the number of powers to include in the model. The quadratic model with a squared interaction term (BIC: 13984) outperformed a quadratic model with only a main effect squared term (BIC: 13988), a linear model with a linear interaction (BIC: 14124), a linear model with no interaction (BIC: 14114), and a model with both squared and cubed terms (BIC: 13999). We also evaluated the relative likelihood of the models (Burnham and Anderson, 2002). We calculated that the most probable model, which is described above, had a model weight of 0.886 (which can be interpreted as the probability of the of the model given the data, the models we evaluated, and a uniform prior over models), using the following formula to calculate model weights for each model *i* (Burnham and Anderson, 2002):

$$weight_{i}=\frac{\exp\text{​}\left(-\left(BIC_{i}-BIC_{min}\right)/2\right)}{\sum_{r}\exp\text{​}\left(-(BIC_{r}-BIC_{min}\right)/2)}$$

The second-most-probable model, which omitted only the squared interaction term, was 0.110 as probable as the selected model. The results from this simplified model were largely similar to those from the more complicated model, though the offset between distractor present and absent trials was significant (β_2_ = 0.20, *p* < 0.001) and the linear interaction term was at trend (β_4_ = 0.006, *p* = 0.06; other terms: β_1_ = −0.09, *p* < 0.0001; β_3_ = 0.003, *p* < 0.0001; no β_5_).

## Results

We measured pupil size and task performance across 72 behavioral sessions conducted with 4 rhesus macaques performing a visual orienting task (Figure 1A) in which social and non-social distractors were presented in a variety of spatial and temporal positions relative to a rewarded target. Both congruent and incongruent distractors influenced response times in this task (Figure 1B; main effect of distractor location, *F*_(3, 38684)_ = 902.97, *p* < 0.05 × 10^−30^). Compared to the baseline response time in the absence of distractors, incongruent distractors slowed response times (paired within-session *t*-test, *p* < 0.01 × 10^−9^) and congruent distractors sped responses (*p* < 0.05 × 10^−8^). Conversely, neutral distractors had little behavioral impact: response times following neutral distractors were not significantly different than response times in the absence of distractors (Figure 1B; paired *t*-test, *p* > 0.05).

We also compared the coefficient of variation in response times (CV; see Methods) following social and non-social distractors across incongruent and congruent distractor locations within each session. The CV provides a measure of the variance in response times both within and between distractor locations, and thus is sensitive to a variety of distractor effects that cannot be detected by differences in response time means alone, such as changes in the interference of a small subset of the heterogeneous set of social images or non-orthodox changes in distractor interference (such as slowed target-detection response times despite a congruent distractor). Replicating previous reports (Ebitz et al., 2013), response time CV was larger for social images than for non-social images [*p* < 0.02, *z*_(71)_ = 2.47, Wilcoxon rank sum; social mean CV = 0.661 ± 0.018 s.e.m., non-social mean CV = 0.611 ± 0.018 s.e.m.; Figure 2C inset].

The presence of distractors also increased the likelihood that a monkey would make an error, either failing to hold target fixation or making a saccade that was not directed toward the targets [paired within-session *t*-test comparing error likelihood in the presence or absence of distractors, *p* < 0.05 × 10^−8^, *t*_(71)_ = 7.25]. Errors were also more likely in the presence of social distractors than non-social distractors [within session paired *t*-test, *p* < 0.0001, *t*_(71)_ = 4.54]. Thus, distractors effectively interfered with performance in this task, and that interference greater for social distractors than for non-social distractors.

We next examined the relationship between baseline pupil size and distractor interference, in terms of the response time and error costs of distractors. Baseline pupil size (average pupil size during the first 350 ms of fixation) predicted an increase in the response time effects of the distractors (Figure 2B; slower responses for incongruent distractors compared to the congruent distractor baseline: *p* < 0.05, β_3_ = 0.0005, though there was no trend toward faster responses for congruent distractors: *p* = 0.7, β_1_ = −0.0001). It also predicted mild slowing of target response times in the absence of distractors (separate GLM analysis, beta = 0.0002, *p* = 0.02), though the reason for this effect is unclear. The relationship between absolute pupil size and distractor interference was roughly doubled when absolute pupil size was measured at distractor presentation. Pupil size at distractor presentation (average from 50 ms before to 50 ms after presentation, before any pupil constriction) also predicted the impact of distractors on response times (incongruent distractors: *p* < 0.0003, β_3_ = 0.0008, trend toward faster responses for congruent distractors: *p* < 0.003, β_1_ = −0.0005). Because foveal luminance was not constant during this period, however, this latter effect should be interpreted with caution.

Baseline pupil size at fixation also predicted the probability of errors, in terms of broken fixations and saccades directed toward distractors rather than the target (Figure 2A). Pupil size had a negative, U-shaped relationship with error rate, regardless of the presence of distractors (slope: *p* < 0.05 × 10^−27^, β_2_ = −0.12, curvature: *p* < 0.04 × 10^−24^, β_3_ = 0.004): errors were most likely when pupil size was either large or small, but were minimized at intermediate pupil sizes. (see Methods for details of model selection procedures). Although distractors evoke a significant global increase in the likelihood of errors in this task [*p* < 0.03 × 10^−21^, *t*_(71)_ = 14.75], no global offset was observed in error likelihood between distractor present and absent trials when baseline pupil size was accounted for by this model [*p* = 0.53, β_1_ = −0.06]. Instead, there was an interaction between baseline pupil size and the likelihood of errors following distractors, with error likelihood increasing non-monotonically with increasing baseline pupil size. Distractors had little impact when pupil size was small, but evoked increased error rates at intermediate and larger pupil sizes, as indicated by a significant change in curvature (*p* < 0.005, β_5_ = −0.002) and slope (*p* < 0.0009, β_4_ = 0.052) in the presence of distractors. A model that contained only an interaction in slope but not in curvature, had a relative model probability of 0.11 (calculated from BIC values, compare to the full model's weight of 0.886; see Methods). In the simpler model, the linear interaction in slope had a non-significant but positive trend (*p* = 0.06, β_4_ = 0.006). Thus, larger baseline pupil size predicted enhanced distractor interference, both in terms of response time and error likelihood.

We next asked whether baseline pupil size predicted a general enhancement in distractibility, or predicted a specific increase in the interference of the biologically salient social distractors. In order to address this question, we determined whether differences in response time CV for social and non-social distractors were modulated by baseline pupil size. Response time CV provided a measure of distractor interference that was sensitive to variance both within and between distractor locations (see Methods) and was modulated by the social content of the distractors (Figure 2C inset). However, the social distractor effect on response time CV was mediated by baseline pupil size (Figure 2C). While there was no significant offset in response time CV for social distractors compared to non-social distractors (*p* = 0.67, β_3_ = −0.006), there was in interaction with baseline pupil size. When baseline pupil size was low, there was little difference between response time CV for social and non-social distractors. However, as baseline pupil size increased, the response time effects of social distractors increased compared to non-social distractors. Response time CV was also globally larger in the presence of distractors (*p* < 0.0001, β_2_ = 0.29) and there was a trend toward a decreasing relationship between pupil size and response time CV in the absence of distractors (*p* = 0.07, β_1_ = −0.029). However, there was also no significant interaction between pupil size and response time CV for non-social distractors compared to the distractor-absent baseline (*p* = 0.56, β_4_ = −0.013).

Because of the mean-normalization inherent in the CV, it remained plausible that these effects were due to systematic changes in the mean response time across both the incongruent and congruent distractor locations, rather than to specific change in the variance of response time following social distractors. Therefore, we next ran the same model on mean response times within each bin, collapsed across both distractor locations when a distractor was present. While we found no significant offset in response time with the presence of distractors (*p* = 0.94, β_4_ = 0.0002), social content predicted a significant increase in mean response time across distractor locations (*p* < 0.02, β_3_ = 0.006), suggesting that social distractors can slow task performance generally, even when physically congruent with the target. As suggested by previous analyses, we also observed slight slowing of response time with increasing baseline pupil size across all three trial types (distractors absent, social, non-social; *p* = 0.03, β_1_ = 0.007). No other effects, including the interaction between social distractor content and baseline pupil size, were significant (*p* > 0.4 for each term; β_2_ = 0.0002, β_4_ = −0.0016, β_5_ = −0.0039). Thus, the interaction between baseline pupil size and the social content of distractors in predicting response time CV cannot be better explained by systematic shifts in the mean response time.

We also examined whether the relationship between error likelihood and baseline pupil size was modulated by the social content of the distractors. Because simply adding additional terms to the original model resulted in a GLM with 9 highly interrelated terms and visibly poor fits to the data, we instead calculated a social distractor error index as the error frequency following social distractors minus error frequency following non-social distractors, normalized by the total number of errors observed within each session. For this analysis, pupil size was evenly divided into 8 bins within session, and the social distractor index was calculated within each bin within each session. The number of bins was selected to maximize the number of bins while still ensuring a small number of missing cells (8 bins: 3 cells with no observed errors; compare 12 empty cells at 9 bins, 2 empty cells at 7 bins). Monkey identity was included as a dummy variable in this analysis.

There was a non-significant trend toward increasing error likelihood for social distractors, relative to non-social distractors, as baseline pupil size increased (*p* = 0.07, beta = 0.002). This trend paralleled the observations for response time CV, again suggesting that baseline pupil size predicts a specific, rather than diffuse, change in attentional priorities.

Next, we characterized the relationship between the pupil light response to distractor onset (hereafter the “pupil distractor response”, see Methods) and vigilance. To ensure that the pupil responses were specific to the distractors and not to other luminance transients in this task, we first determined the relationship between distractor presence and the pupil distractor response. Within each session, pupils were significantly smaller following distractors than in their absence (paired *t*-test, *p* < 0.01 × 10^−25^). We used a ROC analysis to ask how well the maximal constriction in pupil size in the 600 ms following distractor timestamps predicted the presence of a distractor. Within-session area under the curve was consistently high [AUC: mean = 0.86, range = 0.71–0.97; permutation test across sessions, *p* < 0.01 × 10^−46^, *z*_(72)_ = 14.6 all sessions significant, *p* < 0.01], indicating substantial separation in the distributions of pupil traces observed with and without distractors.

Moreover, the pupil distractor response was modulated by the location of the distractor, relative to the target. The pupil distractor response was substantially reduced for neutral distractors compared to either incongruent or congruent distractors [Figure 3A; *p* < 0.0001, *t*_(71)_ = 24.13]. ROC analysis revealed a consistent and reliable relationship between the pupil light response and distractor location across sessions [mean AUC = 0.72, range: 0.53–0.87; 69/72 sessions significant permutation tests, *p* < 0.01; across session Wilcoxon rank sum test, *p* < 0.05 × 10^−46^, *z*_(72)_ = 14.58], suggesting that the pupil light response, like response time effects, was modulated by the distractor's proximity to possible target locations.

Social distractors evoked increased response time interference (Figure 1B) and the response time CV (Figure 2C inset), consistent with enhanced attentional salience of biologically important stimuli. We therefore compared pupil constriction following social distractors to constriction following non-social distractors in all locations. We found that pupil constriction was enhanced for social distractors (Figure 3B), regardless of whether they were in neutral [*p* < 0.05, *t*_(71)_ = −1.80, paired, one tailed *t*-test], incongruent [*p* < 0.01, *t*_(71)_ = −2.56], or congruent locations [*p* < 0.005, *t*_(71)_ = −3.49]. Social content was a significant determinant of pupil size even when controlling for session, SOA, distractor location, baseline pupil size, and response time (*p* < 0.003, beta = −0.002). Thus, the pupil response to distractors was modulated by whether they were social images.

Pupil responses were also correlated with the level of distractor interference within trials (Figure 3C, bars ± s.e.m.). As pupil responses increased in magnitude, response times slowed for incongruent distractors relative to the congruent baseline (*p* < 0.0001, β_3_ = 0.001) and shortened for congruent distractors (*p* < 0.0001, β_1_ = −0.003). This effect was not better explained by differences in the pupil light response across SOA bins (Figure 4). When we only examined response time following distractors presented before target onset (SOA <0), we observed the same effects (β_1_ = −0.0005, *p* < 0.004; β_2_ = 0.09, *p* < 0.0001; β_3_ = 0.002, *p* < 0.0001). Moreover, when SOAs were equated, the interaction term was roughly doubled in magnitude (equated SOA β_3_ = 0.002; across SOAs β_3_ = 0.001), suggesting that SOA differences did not explain, but rather complicated this relationship. This finding refutes the hypothesis that visual field inhomogeneity can explain the modulation of the pupil distractor response by distractor congruency. Moreover, the observation that the pupil response predicted both slowed response time for incongruent distractors and sped response time for congruent distractors indicates that the response time effects were not due to any difficulty in target detection, but rather reflect the level of interference of the distractors on task performance.

One possible interpretation of these findings is that differences in autonomic arousal and baseline pupil size could explain both the variance in distractor interference and the variance in the pupil light response. Changes in baseline pupil size could have introduced floor or ceiling effects due to the physiological limits on absolute pupil size. Alternatively, arousal may have influenced both baseline pupil size and the magnitude of the pupil light response. The threat of shock, for example, both increases baseline pupil size and reduces the pupil light response (Bitsios et al., 1996). Therefore, we next determined whether differences in baseline pupil size predicted the pupil light response and its modulation by dynamic changes in attention.

Although baseline pupil size was modestly predictive of the response time effects of distractors, it did not mediate the observed relationship between the pupil light response and distractor interference. While larger initial pupil size was associated with a constant decrease in pupil size following distractor onset (*p* < 0.0001, β_1_ = −0.0003), we observed no baseline-dependent changes in the pupil light response to incongruent distractors (*p* = 0.19, β_3_ = 0.006), though there was a significant interaction for congruent distractors (*p* = 0.05, β_3_ = −0.01). Moreover, response times were correlated with the pupil light response after controlling for baseline pupil size (Figures 3D,E). No interaction was observed between baseline pupil size and response times for incongruent trials (*p* > 0.73, β_5_ = 0.0003), though a small interaction was observed within congruent trials (*p* < 0.03, β_5_ = −0.002). Overall, however, there was no systematic change in the relationship between distractor interference and the pupil light response across baseline pupil size. To confirm this interpretation, we also controlled for the relationship between baseline pupil size and the pupil light response by stepwise regression (see Methods). However, increasing pupil light response magnitudes still predicted slower responses following incongruent distractors (*p* < 0.002, β_3_ = 0.0005) and faster responses following congruent distractors (*p* < 0.0003, β_1_ = −0.0008). Thus, while baseline pupil size predicted both reaction times following distractors and the magnitude of the pupil light response, it did not mediate the relationship between these two measures.

We next asked whether the pupil light response was modulated by saccade preparation (Figure 4). Attention, as indexed by visual discrimination, is directed toward the location of impending saccades (Hoffman and Subramaniam, 1995; Kowler et al., 1995). Therefore, we compared pupil light responses to distractors presented before and after target onset, located either congruent or incongruent with respect to the saccade target. The pupil response was identical for congruent and incongruent distractors presented before target onset [Figure 4A; *p* > 0.86, *t*_(71)_ = 0.18; mean AUC across sessions = 0.49, range = 0.36–0.66]. Nevertheless, when distractors were presented after the target appeared, pupil constriction was increased for congruent distractors compared to incongruent distractors [Figure 4B; *p* < 0.0003, *t*_(71)_ = 3.79; mean AUC = 0.63, range = 0.41–0.81]. This effect was due to both enhanced pupil responses for congruent distractors, which were proximal to the saccade target, and suppressed responses to incongruent distractors [Figure 4C, bars ± s.e.m.; interaction of SOA bin and distractor congruence, *p* < 0.0001, *F*_(5, 20258)_ = 19.3]. Pupil responses were suppressed for all distractors that immediately followed the target (*p* < 0.05), perhaps due to attentional blink, but at longer SOAs, responses to congruent distractors were enhanced (*p* < 0.05) and responses to incongruent distractors were suppressed (*p* < 0.05). The main effect of distractor congruence in this analysis [*p* < 0.0001, *F*_(1, 20258)_ = 89.2] was driven by post-target distractors (*p* < 0.05). Thus, the pupil light response was enhanced when planning a gaze shift toward targets in the same hemifield as distractors and suppressed when planning a gaze shift away from distractors.

## Discussion

Pupil size under constant luminance is correlated with the activity of neurons in the locus coeruleus (Gilzenrat et al., 2010) and is a commonly used index of NE tone (Samuels and Szabadi, 2008; Gilzenrat et al., 2010; Jepma and Nieuwenhuis, 2011; Nassar et al., 2012; Eldar et al., 2013). NE has long been hypothesized to be a potent determinant of task performance and distractibility (Aston-Jones and Cohen, 2005; Sara and Bouret, 2012), but empirical support for this idea has been elusive (Carli et al., 1983; Clark et al., 1989; Witte and Marrocco, 1997). Here, we report that baseline pupil size predicts dynamic changes in distractibility, as indexed by the impact of distractors on both response times and error rates, consistent with the hypothesis that NE regulates the balance of distractibility and focus. However, in contrast to a generalized distractibility hypothesis (Aston-Jones and Cohen, 2005; Sara and Bouret, 2012), the pupil-liked change in distractor interference was a specific sharpening of attention toward the most biologically important distractors in this study. Task-irrelevant faces outcompete task relevant targets for attention (Cerf et al., 2009; Ebitz et al., 2013), but here we show that the interference of faces was modulated by baseline pupil size.

From an adaptive perspective, this makes a great deal of sense. When arousal is high, as it is in the presence of threat, it is maladaptive for attention to be truly labile, captured by any stimulus regardless of its relevance to the threat. Ideally, attention should instead sharpen toward the most threat-relevant stimuli, regardless of ongoing goals or other sources of distraction. Though it remains unclear whether vigilance for other stimuli is also modulated by baseline pupil size, our data show that baseline pupil size can predict specific, rather than general shifts in distractibility. Thus, these data endorse the hypothesis that pupil-linked arousal mechanisms such as NE are involved in the regulation of vigilance for stimuli that are salient for the animal, such as the faces of other individuals.

The pupil light response is not entirely reflexive, but the cognitive and cortical processes that influence it remain poorly understood. Here, we report that the pupil light response varies with dynamic changes in attention on both long and short time scales; the pupil response varied with the magnitude of the pupil under constant luminance conditions, but also independently scaled with distractor attention. Within trials, the magnitude of the pupil light response varied with saccade preparation, distractor congruence, and the social significance of distractors. These findings compliment and extend previous observations that the magnitude of the pupil light response is influenced by attentional cues (Binda et al., 2013a) and stimulus awareness (Hakerem and Sutton, 1966; Zuber et al., 1966), even in the absence of a luminance increment (Binda et al., 2013b). However, in those previous studies, attention and/or stimulus awareness were confounded with effort and arousal, because the stimuli that elicited the pupil light response were task-relevant. Because we used task-irrelevant distractors, increasing effort in this task would reduce distractor interference. Yet, the magnitude of the pupil light response scaled positively with distractor interference. Our findings thus suggest that the pupil light response tracks dynamic changes in attention, rather than effort or arousal.

As a peripheral, physiological index of vigilance, the pupil light response has potential utility both in the lab and in human-machine interfaces. Measuring vigilance currently relies principally on behavioral metrics such as response time interference, which cannot be acquired in every task. Here, we show that simply measuring pupil constriction in response to distractors can effectively substitute for response time metrics as a measure of a trial-by-trial level of distraction and may even be an improvement over these metrics, due to relative immunity to the influence of pupil-linked arousal. Combining this observation with the deconvolution methods recently developed for interpreting continuous pupil size measurements (Wierda et al., 2012) may prove particularly powerful.

Pupil size also has consequences for visual perception, though how these optical effects shape attention and visual behavior remain poorly understood. Larger pupil size, for example, increases spherical aberrations, and could thereby increase the difficulty of detecting a small target. In our study, larger baseline pupil size predicted slowed response times in the absence of distractors, which might reflect difficulty detecting the target. However, these effects were accompanied by reduced, rather than increased, error rates, suggesting that baseline pupil size may predict changes in speed-accuracy tradeoff rather than difficulty in target perception *per se*. It is also possible that changes in baseline pupil size may affect distractor perception by enhancing visual salience. In particular, larger baseline pupil size would defocus the visual scene, thereby limiting the resolution of high spatial frequencies needed to perceive edges and texture, which would otherwise draw attention during natural image viewing (Itti and Koch, 2001). Thus, regulating pupil size may be a simple mechanism that biases visual scanning away from high spatial frequencies and toward other visual features such as movement or high contrast features in the low spatial frequency domain.

In parallel, enhanced pupil light responses would have many of the same perceptual consequences as attention. Reduced pupil diameter necessarily improves visual acuity and contrast sensitivity by decreasing defocus and reducing spherical aberrations. These optical effects cannot fully explain the perceptual effects of attention (Yeshurun and Carrasco, 1998; Carrasco et al., 2002, 2004). For example, attention can modulate contrast sensitivity of neurons within a particular retinotopic location in extrastriate cortex without affecting contrast sensitivity to a second location (Reynolds et al., 2000). A global change in pupil diameter could not explain this effect. The perceptual consequences of attention and pupil size also differ in magnitude. Attention improves visual acuity on the order of several arc minutes (Carrasco et al., 2002). For individuals with normal (20/20) vision, the change in pupil size that would be required to produce the equivalent change in visual acuity would be larger than the physiological range of the pupil (Atchison et al., 1979). Nevertheless, in even mildly myopic individuals, the perceptual effects of 1 mm reductions in pupil diameter can produce arc minute changes in visual acuity (Atchison et al., 1979). Similarly, defocus may have more profound effects on contrast sensitivity than on visual acuity (Rabin, 1994), so by extension, the effects of the pupil light response on contrast sensitivity may be more pronounced. Critically, the methods used to measure visual acuity and myopia differ between the opthamology clinic and the lab, so it is difficult to directly compare these observations. Future work will be needed to fully understand the perceptual consequences of both attention and pupil size. At minimum, attentional modulation of the pupil response may work synergistically with other mechanisms to shape the perceptual effects of attention.

In addition to global differences in luminance, natural environments include local gradients in luminance. Thus, two sequential saccades can target regions that evoke very different pupil diameters. For example, one might shift gaze away from a dimly lit desk to a bright window. The pre-saccadic modulation of the pupil light response reported here may permit anticipatory adjustments in pupil size in preparation for upcoming saccades. The pupil requires hundreds of milliseconds to constrict to its minimal size following a light stimulus (Clarke et al., 2003). Initializing constriction before saccade onset would give the pupil time to reach optimal size before the target is foveated. This process would reduce retinal fatigue and improve target signal during viewing of natural scenes with local luminance gradients, potentially improving scanning efficiency. Moreover, there is anatomical evidence for oculomotor modulation of the pupil light response. The pupil light reflex is mediated by a subcortical pathway from the retina, through the Edinger-Westphal nucleus and pretectum, to the ciliary ganglia that constrict the pupil. However, a small pupil light response is observed in the absence of direct retinal input to pretectum (Papageorgiou et al., 2008), suggesting other inputs to this pathway. That input may arise from the projections the pretectum receives from regions critical for oculomotor processes (Gamlin, 2006), including the lateral intraparietal cortex (Asanuma et al., 1985) and the frontal eye fields (Künzle and Akert, 1977; Leichnetz, 1982; Huerta et al., 1986). Future work will be needed to test this hypothesis empirically.

In summary, we report that the pupil indexes both the state of species-typical vigilance and dynamic changes in attention during performance of a social vigilance task. These observations introduce a novel behavioral metric of attention, in the pupil distractor response, that may prove useful as an implicit, peripheral metric of social attention. Moreover, these observations enhance our understanding of the state of vigilance. When arousal is high, attention is not simply labile, but rather may be focused on those stimuli with the most biological relevance. This result highlights the importance of situating task performance in a naturalistic setting. Failures of task-performance can be due to failures of goal states, but they can also be due to the organism's endogenous and species-typical priorities, which compete with task-relevant goals for expression.

## Author contributions

R. Becket Ebitz and Michael L. Platt designed the experiments. R. Becket Ebitz, Michael L. Platt, and John M. Pearson wrote the manuscript. R. Becket Ebitz collected the data. R. Becket Ebitz and John M. Pearson analyzed the data.

### Conflict of interest statement

The authors declare that the research was conducted in the absence of any commercial or financial relationships that could be construed as a potential conflict of interest.

## References

[B1] Abrams R. Christ S. (2003). Motion onset captures attention. Psychol. Sci. 14, 427–432 10.1111/1467-9280.01458 12930472

[B2] Arnsten A. F. (2009). Stress signalling pathways that impair prefrontal cortex structure and function. Nat. Rev. Neurosci. 10, 410–422 10.1038/nrn2648 19455173 PMC2907136

[B3] Asanuma C. Andersen R. A. Cowan W. M. (1985). The thalamic relations of the caudal inferior parietal lobule and the lateral prefrontal cortex in monkeys: divergent cortical projections from cell clusters in the medial pulvinar nucleus. J. Comp. Neurol. 241, 357–381 10.1002/cne.902410309 4086661

[B4] Aston-Jones G. Cohen J. D. (2005). An integrative theory of locus coeruleus-norepinephrine function: adaptive gain and optimal performance. Annu. Rev. Neurosci. 28, 403–450 10.1146/annurev.neuro.28.061604.135709 16022602

[B5] Aston-Jones G. Rajkowski J. Cohen J. (1999). Role of locus coeruleus in attention and behavioral flexibility. Biol. Psychiatry 46, 1309–1320 10.1016/S0006-3223(99)00140-7 10560036

[B6] Atchison D. A. Smith G. Efron N. (1979). The effect of pupil size on visual acuity in uncorrected and corrected myopia. Am. J. Optom. Physiol. Opt. 56, 315–323 10.1097/00006324-197905000-00006 495689

[B7] Barbur J. L. Harlow A. J. Sahraie A. (1992). Pupillary responses to stimulus structure, colour and movement. Ophthalmic Physiol. Opt. 12, 137–141 10.1111/j.1475-1313.1992.tb00276.x 1408159

[B8] Binda P. Pereverzeva M. Murray S. O. (2013a). Attention to bright surfaces enhances the pupillary light reflex. J. Neurosci. 33, 2199–2204 10.1523/JNEUROSCI.3440-12.2013 23365255 PMC6619119

[B9] Binda P. Pereverzeva M. Murray S. O. (2013b). Pupil constrictions to photographs of the sun. J. Vis. 13, 8.1–8.9 10.1167/13.7.13 23685391

[B10] Bitsios P. Szabadi E. Bradshaw C. (1996). The inhibition of the pupillary light reflex by the threat of an electric shock: a potential laboratory model of human anxiety. J. Psychopharmacol. 12, 137–145 22302974 10.1177/026988119601000404

[B11] Bitsios P. Szabadi E. Bradshaw C. (1998). The effects of clonidine on the fear-inhibited light reflex. J. Psychopharmacol. 12, 137–145 10.1177/026988119801200204 9694025

[B12] Bunsey M. Strupp B. (1995). Specific effects of idazoxan in a distraction task: Evidence that endogenous norepinephrine plays a role in selective attention in rats. Behav. Neurosci. 109, 903 10.1037/0735-7044.109.5.903 8554714

[B13] Burnham K. P. Anderson D. R. (2002). Model Selection and Multi-model Inference: A Practical Information-Theoretic Approach, 2nd Edn., New York, NY: Springer

[B14] Carli M. Robbins T. W. Evenden J. L. Everitt B. J. (1983). Effects of lesions to ascending noradrenergic neurones on performance of a 5-choice serial reaction task in rats; implications for theories of dorsal noradrenergic bundle function based on selective attention and arousal. Behav. Brain Res. 9, 361–380 10.1016/0166-4328(83)90138-9 6639741

[B15] Carrasco M. Ling S. Read S. (2004). Attention alters appearance. Nat. Neurosci. 7, 308–313 10.1038/nn1194 14966522 PMC3882082

[B16] Carrasco M. Williams P. E. Yeshurun Y. (2002). Covert attention increases spatial resolution with or without masks: support for signal enhancement. J. Vis. 2, 467–479 10.1167/2.6.4 12678645

[B17] Cerf M. Frady E. P. Koch C. (2009). Faces and text attract gaze independent of the task: Experimental data and computer model. J. Vis. 9, 10.1–10.15 10.1167/9.12.10 20053101

[B18] Clark C. R. Geffen G. M. Geffen L. B. (1989). Catecholamines and the covert orientation of attention in humans. Neuropsychologia 27, 131–139 10.1016/0028-3932(89)90166-8 2538773

[B19] Clarke R. J. Zhang H. Gamlin P. D. R. (2003). Characteristics of the pupillary light reflex in the alert rhesus monkey. J. Neurophysiol. 89, 3179–3189 10.1152/jn.01131.2002 12611973

[B20] Ebitz R. B. Watson K. K. Platt M. L. (2013). Oxytocin blunts social vigilance in the rhesus macaque. Proc. Natl. Acad. Sci. U.S.A. 110, 11630–11635 10.1073/pnas.1305230110 23798448 PMC3710816

[B21] Eldar E. Cohen J. D. Niv Y. (2013). The effects of neural gain on attention and learning. Nat. Neurosci. 16, 1146–1153 10.1038/nn.3428 23770566 PMC3725201

[B22] Foote S. L. Aston-Jones G. Bloom F. E. (1980). Impulse activity of locus coeruleus neurons in awake rats and monkeys is a function of sensory stimulation and arousal. Proc. Natl. Acad. Sci. U.S.A. 77, 3033–3037 10.1073/pnas.77.5.3033 6771765 PMC349541

[B23] Gamlin P. Zhang H. Harlow A. Barbur J. (1998). Pupil responses to stimulus color, structure and light flux increments in the rhesus monkey. Vision Res. 38, 3353–3358 10.1016/S0042-6989(98)00096-0 9893848

[B24] Gamlin P. D. R. (2006). The pretectum: connections and oculomotor-related roles. Prog. Brain Res. 151, 379–405 10.1016/S0079-6123(05)51012-4 16221595

[B25] Gilzenrat M. S. Nieuwenhuis S. Jepma M. Cohen J. D. (2010). Pupil diameter tracks changes in control state predicted by the adaptive gain theory of locus coeruleus function. Cogn. Affect. Behav. Neurosci. 10, 252–269 10.3758/CABN.10.2.252 20498349 PMC3403821

[B26] Hakerem G. Sutton S. (1966). Pupillary response at visual threshold. Nature 212, 485–486 10.1038/212485a0 5970183

[B27] Hayden B. Y. Nair A. C. McCoy A. N. Platt M. L. (2008). Posterior cingulate cortex mediates outcome-contingent allocation of behavior. Neuron 60, 19–25 10.1016/j.neuron.2008.09.012 18940585 PMC2575690

[B28] Hirsch B. (2002). Social monitoring and vigilance behavior in brown capuchin monkeys (*Cebus apella*). Behav. Ecol. Sociobiol. 52, 458–464 10.1007/s00265-002-0536-5

[B29] Hoffman J. E. Subramaniam B. (1995). The role of visual attention in saccadic eye movements. Percept. Psychophys. 57, 787–795 10.3758/BF03206794 7651803

[B30] Huerta M. F. Krubitzer L. A. Kaas J. H. (1986). Frontal eye field as defined by intracortical microstimulation in squirrel monkeys, owl monkeys, and macaque monkeys: I. Subcortical connections. J. Comp. Neurol. 253, 415–439 10.1002/cne.902530402 3793998

[B31] Hunter L. Skinner J. (1998). Vigilance behaviour in African ungulates: the role of predation pressure. Behaviour 135, 195–211 10.1163/156853998793066320

[B32] Itti L. Koch C. (2001). Computational modelling of visual attention. Nat. Rev. Neurosci. 2, 194–203 10.1038/35058500 11256080

[B33] Jepma M. Nieuwenhuis S. (2011). Pupil diameter predicts changes in the exploration–exploitation trade-off: evidence for the adaptive gain theory. J. Cogn. Neurosci. 23, 1587–1596 10.1162/jocn.2010.21548 20666595

[B34] Kardon R. (1995). Pupillary light reflex. Curr. Opin. Ophthalmol. 6, 20–26 10.1097/00055735-199512000-00004 10160414

[B35] Kowler E. Anderson E. Dosher B. Blaser E. (1995). The role of attention in the programming of saccades. Vision Res. 35, 1897–1916 10.1016/0042-6989(94)00279-U 7660596

[B36] Künzle H. Akert K. (1977). Efferent connections of cortical, area 8 (frontal eye field) in *Macaca fascicularis*. A reinvestigation using the autoradiographic technique. J. Comp. Neurol. 173, 147–164 10.1002/cne.901730108 403205

[B37] Lazarus J. (1978). Vigilance, flock size and domain of danger size in the white-fronted goose. Wildfowl 29, 135–145

[B38] Leichnetz G. R. (1982). Connections between the frontal eye field and pretectum in the monkey: an anterograde/retrograde study using HRP gel and TMB neurohistochemistry. J. Comp. Neurol. 207, 394–404 10.1002/cne.902070410 7119150

[B39] Nassar M. R. Rumsey K. M. Wilson R. C. Parikh K. Heasly B. Gold J. I. (2012). Rational regulation of learning dynamics by pupil-linked arousal systems. Nat. Neurosci. 15, 1040–1046 10.1038/nn.3130 22660479 PMC3386464

[B40] Papageorgiou E. Ticini L. F. Hardiess G. Schaeffel F. Wiethoelter H. Mallot H. A. (2008). The pupillary light reflex pathway: cytoarchitectonic probabilistic maps in hemianopic patients. Neurology 70, 956–963 10.1212/01.wnl.0000305962.93520.ed 18347318

[B41] Pöysä H. (1994). Group foraging, distance to cover and vigilance in the teal, *Anas crecca*. Anim. Behav. 48, 921–928 10.1006/anbe.1994.1317

[B42] Rabin J. (1994). Optical defocus: differential effects on size and contrast letter recognition thresholds. Invest. Ophthalmol. Vis. Sci. 35, 646–648 8113015

[B43] Rajkowski J. Kubiak P. Aston-Jones G. (1994). Locus coeruleus activity in monkey: Phasic and tonic changes are associated with altered vigilance. Brain Res. Bull. 35, 607–616 10.1016/0361-9230(94)90175-9 7859118

[B44] Reynolds J. H. Pasternak T. Desimone R. (2000). Attention increases sensitivity of V4 neurons. Neuron 26, 703–714 10.1016/S0896-6273(00)81206-4 10896165

[B45] Roberts G. (1996). Why individual vigilance declines as group size increases. Anim. Behav. 51, 1077–1086 10.1006/anbe.1996.0109

[B46] Sahraie A. Barbur J. L. (1997). Pupil response triggered by the onset of coherent motion. Graefes Arch. Clin. Exp. Ophthalmol. 235, 494–500 10.1007/BF00947006 9285218

[B47] Samuels E. Szabadi E. (2008). Functional neuroanatomy of the noradrenergic locus coeruleus: its roles in the regulation of arousal and autonomic function part II: physiological and pharmacological manipulations and pathological alterations of locus coeruleus activity in humans. Curr. Neuropharmacol. 6, 254 10.2174/157015908785777193 19506724 PMC2687931

[B48] Sara S. J. Bouret S. (2012). Orienting and reorienting: the locus coeruleus mediates cognition through arousal. Neuron 76, 130–141 10.1016/j.neuron.2012.09.011 23040811

[B49] Steinhauer S. R. Condray R. Kasparek A. (2000). Cognitive modulation of midbrain function: task-induced reduction of the pupillary light reflex. Int. J. Psychophysiol. 39, 21–30 10.1016/S0167-8760(00)00119-7 11120344

[B50] Wierda S. M. Van Rijn H. Taatgen N. A. Martens S. (2012). Pupil dilation deconvolution reveals the dynamics of attention at high temporal resolution. Proc. Natl. Acad. Sci. U.S.A. 109, 8456–8460 10.1073/pnas.1201858109 22586101 PMC3365158

[B51] Witte E. A. Marrocco R. T. (1997). Alteration of brain noradrenergic activity in rhesus monkeys affects the alerting component of covert orienting. Psychopharmacology 132, 315–323 10.1007/s002130050351 9298508

[B52] Yeshurun Y. Carrasco M. (1998). Attention improves or impairs visual performance by enhancing spatial resolution. Nature 396, 72–75 10.1038/23936 9817201 PMC3825508

[B53] Yu A. J. Dayan P. (2005). Uncertainty, neuromodulation, and attention. Neuron 46, 681–692 10.1016/j.neuron.2005.04.026 15944135

[B54] Zuber B. L. Stark L. Lorber M. (1966). Saccadic suppression of the pupillary light reflex. Exp. Neurol. 14, 351–370 10.1016/0014-4886(66)90120-8 4951848

